# Explainable machine learning for predicting in-hospital mortality in acute heart failure: a prospective single-center cohort study from Vietnam

**DOI:** 10.3389/fcvm.2026.1927124

**Published:** 2026-10-21

**Authors:** Sy Van Hoang, Kha Minh Nguyen, Tai Nhat Nguyen, Huy Nhat Do

**Affiliations:** 1 Department of Internal Medicine, School of Medicine, University of Medicine and Pharmacy at Ho Chi Minh City, Ho Chi Minh City, Vietnam; 2 Department of Cardiology, Cho Ray Hospital, Ho Chi Minh City, Vietnam; 3 Department of Cardiology, Nhan Dan Gia Dinh Hospital, Ho Chi Minh City, Vietnam

**Keywords:** acute heart failure, explainable artificial intelligence, GWTG-HF score, in-hospital mortality, machine learning, risk prediction, SHAP

## Abstract

**Background:**

Accurate risk stratification is essential in acute heart failure (AHF), which carries substantial in-hospital mortality. The Get With The Guidelines–Heart Failure (GWTG-HF) score is widely used, but its performance may vary across populations, and prospective data from Southeast Asia are scarce. We developed and internally validated explainable machine-learning (ML) models for in-hospital mortality in a Vietnamese AHF cohort and compared them with the GWTG-HF score.

**Methods:**

We prospectively enrolled 572 consecutive patients admitted with AHF (October 2024–June 2025). Missing data were imputed with a random forest–based algorithm, and class imbalance handled with SMOTE within training folds. Six algorithms (logistic regression, elastic net, random forest, XGBoost, LightGBM, CatBoost) were trained with stratified cross-validation, with optimism correction, and evaluated on an independent 25% test set. We assessed discrimination (ROC-AUC, PR-AUC), calibration (calibration slope/intercept, Brier and scaled Brier scores), classification, and clinical utility (decision-curve analysis); thresholds were derived from training data only. Pairwise AUC comparisons used the DeLong test, and interpretation used SHAP. Reporting followed TRIPOD + AI.

**Results:**

Fifty-three patients (9.3%) died in hospital. Random forest had the highest discrimination (AUC 0.846, 95% CI 0.757–0.922), followed by the GWTG-HF score (0.817), logistic regression (0.795), elastic net (0.794), CatBoost (0.786), XGBoost (0.763), and LightGBM (0.694). No ML model significantly outperformed the GWTG-HF score (all DeLong *p* > 0.05), a comparison limited by only 13 test-set events. Although CatBoost had the numerically lowest Brier score (0.085), scaled Brier scores were low for all models and calibration intercepts were uniformly negative, indicating over-estimation of risk from aggressive imbalance correction; predicted probabilities therefore require recalibration. SHAP identified respiratory failure, NT-proBNP, high-sensitivity troponin I (risk-increasing) and an ADHF phenotype and left ventricular dilation (associated with lower predicted risk) as the most influential features.

**Conclusions:**

Explainable ML, led by random forest, achieved good discrimination but did not significantly outperform the GWTG-HF score, most plausibly reflecting limited statistical power rather than true equivalence. With miscalibrated probabilities requiring recalibration, explainable ML is best viewed as a complementary risk-ranking tool; external, multicenter, and temporal validation are required before clinical use.

## 1 Introduction

Heart failure is a complex clinical syndrome that affects more than 60 million people worldwide and imposes a growing burden on health systems (1). Acute heart failure (AHF) is among the leading causes of unplanned hospitalization, accounting for approximately 1%–2% of all admissions in high-income countries, and in-hospital mortality remains substantial at 4%–10% despite advances in diagnostic and therapeutic strategies (2, 3). This burden is particularly relevant in Asia, where registry data show that patients from Southeast Asia experience higher one-year mortality than those from other Asian regions, underscoring important regional differences in disease profile and outcomes (4). Early and accurate risk stratification is therefore pivotal for guiding the intensity of monitoring and treatment and for improving clinical outcomes (5).

Several prognostic tools have been developed for hospitalized heart failure, ranging from the classification-and-regression-tree model derived from the ADHERE registry (6) to the Get With The Guidelines–Heart Failure (GWTG-HF) risk score, one of the most widely adopted instruments (7, 8). Most of these models are built on conventional logistic regression, which assumes additive and largely linear relationships between predictors and outcome and may therefore fail to capture the complex interactions and nonlinear patterns inherent in clinical data (9). Moreover, the discrimination of existing scores is only moderate in some populations, which limits their generalizability across diverse settings (8).

Artificial intelligence, and machine learning (ML) in particular, has advanced rapidly across cardiovascular medicine, with applications spanning diagnosis, prognosis, risk stratification, medical imaging, and clinical decision support (10, 11). ML models can accommodate high-dimensional data and learn nonlinear relationships and higher-order interactions among variables, potentially improving predictive performance relative to conventional statistical methods (12). In heart failure specifically, algorithms such as random forest, extreme gradient boosting, and deep neural networks have shown strong performance for in-hospital mortality and readmission (13–15). This advantage is not universal, however: a systematic review found no consistent performance benefit of ML over logistic regression for clinical prediction models, especially in studies at low risk of bias (16). Whether ML confers incremental value therefore depends heavily on the clinical context, the data structure, and the number of outcome events.

Three gaps motivated this work. First, many existing ML studies were developed from administrative databases, intensive-care cohorts, or large retrospective registries that may not reflect the real-world spectrum of patients hospitalized with AHF. Second, prospective evidence from low- and middle-income countries remains scarce, particularly in Southeast Asia, where differences in disease profile, healthcare organization, and treatment practices may affect model performance and generalizability (4). Third, concerns about model transparency continue to limit the clinical implementation of ML; the ability to explain how individual predictors contribute to a prediction is increasingly regarded as a prerequisite for trustworthy and clinically applicable systems.

Accordingly, the primary contribution of this study is not a methodological advance but a prospective, real-world regional validation and head-to-head comparison of explainable ML against an established clinical score in an underrepresented Southeast Asian population. Our objectives were (i) to develop and internally validate multiple ML models for predicting in-hospital mortality; (ii) to benchmark their discrimination, calibration, and clinical utility against the GWTG-HF risk score; and (iii) to improve interpretability through SHapley Additive exPlanations (SHAP). The study was designed and is reported in accordance with the TRIPOD + AI statement (17).

## 2 Materials and methods

### 2.1 Study design, population, and prediction time point

This prospective observational cohort study was conducted at the Department of Cardiology, Cho Ray Hospital, a tertiary referral center in southern Vietnam, from October 2024 to June 2025. Consecutive patients aged ≥18 years admitted with a diagnosis of AHF according to the 2021 European Society of Cardiology (ESC) criteria were screened (2). Exclusion criteria were (i) isolated right ventricular failure; (ii) refusal to participate; and (iii) inability to ascertain the outcome, including patients discharged against medical advice in critical condition without reliable follow-up; for the latter, survival within 24 h of discharge was verified by direct family contact. The primary endpoint was all-cause in-hospital mortality during the index hospitalization. The prediction (index) time point was hospital admission: all candidate predictors were ascertained at admission or within the first 24 h and before the outcome, precluding temporal or outcome-related leakage. Presenting features such as respiratory failure and cardiogenic shock were defined by their status at initial presentation rather than by events occurring later during hospitalization.

### 2.2 Data collection

Clinical data were collected prospectively using standardized case-report forms and comprised demographic characteristics, medical history, comorbidities, presenting clinical features, vital signs, laboratory findings, electrocardiographic parameters, and echocardiographic measurements obtained at admission or during the earliest phase of hospitalization. Laboratory variables included complete blood count, serum electrolytes, renal and liver function tests, cardiac biomarkers, and N-terminal pro–B-type natriuretic peptide (NT-proBNP). For NT-proBNP values exceeding the assay upper limit (>35,000 pg/mL), a value of 35,001 pg/mL was assigned to preserve numerical continuity.

### 2.3 GWTG-HF risk score

The GWTG-HF risk score was calculated for each patient using the original validated model, incorporating age, systolic blood pressure, heart rate, blood urea nitrogen, serum sodium, chronic obstructive pulmonary disease, and race (7). Given the homogeneous Asian population, all patients were categorized as non-Black for the race component, consistent with previous validation studies in non-African populations (18, 19).

### 2.4 Data preprocessing, candidate predictors, and sample size

Variables with more than 20% missing values were excluded (systolic pulmonary artery pressure). The dataset was then divided into a training set (75%, *n* = 429) and an independent test set (25%, *n* = 143) using sampling stratified by in-hospital mortality. To avoid leakage, all preprocessing was fitted on the training set only and applied to the test set. Missing values were imputed using a random forest–based iterative algorithm (20); categorical variables were label-encoded and continuous variables standardized. After encoding, 31 candidate predictors entered the models. No *a priori* feature selection was performed, in order to preserve potential nonlinear interactions; instead, pairwise correlations and variance inflation factors were examined to characterize multicollinearity (Supplementary Table S2), and embedded regularization and tree-based selection reduced the effective dimensionality. With 53 events and 31 candidate predictors, the events-per-variable (EPV) ratio was approximately 1.7 (1.3 in the development set); this is below conventional thresholds and the minimum sample size recommended for prediction-model development (21). We therefore prioritized parsimonious, regularized models and quantified overfitting through the optimism-correction procedures described below, and we discuss the implications for model stability explicitly.

### 2.5 Handling class imbalance

Because in-hospital mortality was imbalanced (9.3%), the synthetic minority over-sampling technique (SMOTE) was applied exclusively within each cross-validation training fold (22), and class-weighting was incorporated into applicable algorithms. Because simultaneously combining SMOTE, class weighting, and the scale_pos_weight parameter can over-correct imbalance and distort predicted probabilities (23), we performed a pre-specified sensitivity analysis comparing (i) no imbalance correction, (ii) a single strategy, and (iii) the combined approach, and we report their effect on both discrimination and calibration (Supplementary Table S3).

### 2.6 Model development, hyperparameters, and reproducibility

Six models were developed: logistic regression, elastic-net logistic regression, random forest (24), extreme gradient boosting (XGBoost) (25), light gradient boosting machine (LightGBM) (26), and categorical boosting (CatBoost) (27). Hyperparameters were selected by grid/random search over predefined, deliberately conservative ranges (shallow trees, reduced learning rates, subsampling, and minimum-leaf constraints), optimizing the cross-validated AUC. To ensure reproducibility, the complete search spaces, selected values, optimization criterion, random seeds, and software/library versions for every algorithm are reported in Supplementary Table S1.

### 2.7 Internal validation and optimism correction

In addition to the single stratified 75/25 split with 10-fold cross-validation within the training set, we performed repeated (10 × 10) stratified cross-validation and 1,000-replicate bootstrap resampling to obtain optimism-corrected estimates of discrimination and calibration; nested cross-validation was used wherever hyperparameters were tuned, so that model selection did not contaminate performance estimation (28). Temporal and multicenter external validation were not feasible within this single-center, nine-month prospective cohort and are identified as priorities for future work.

### 2.8 Threshold optimization

Because the clinical priority was to identify high-risk patients, probability thresholds were derived exclusively from the training/cross-validation predictions using a sensitivity-oriented strategy (target sensitivity ≥0.80 while maximizing specificity; otherwise maximizing the F2-score) and were then applied unchanged to the test set. Test-set outcomes did not influence threshold selection; consequently, the reported classification metrics are not optimistically biased by threshold tuning.

### 2.9 Performance evaluation

Discrimination was summarized by the ROC-AUC with 95% confidence intervals (CIs). Given the class imbalance, we additionally report the area under the precision–recall curve (PR-AUC) (29). Classification metrics (sensitivity, specificity, precision, negative predictive value, F1, F2, accuracy) are reported with 95% CIs (Wilson method for proportions). Calibration was assessed with calibration plots (with 95% CIs), the calibration intercept and slope, the Brier score, and the scaled Brier score (Brier skill score) referenced to the no-information Brier score of p(1−p) (23, 28). The GWTG-HF score was evaluated with the same discrimination and calibration metrics and, after applying its threshold, the same classification metrics, to ensure a fair comparison. Pairwise AUC comparisons used the DeLong test (30), and clinical utility was assessed by decision-curve analysis across a range of threshold probabilities (31).

### 2.10 Explainability and model selection

The model taken forward for SHAP interpretation was pre-specified as the algorithm with the best cross-validated discrimination in the training data; random forest met this criterion and also achieved the highest AUC on the independent test set (32). Global feature importance was quantified by mean absolute SHAP values, and SHAP summary, dependence, and local-explanation plots were generated. For binary and ordinal predictors, the direction of the SHAP contribution (protective vs. risk-increasing) was verified against the observed univariable associations.

### 2.11 Statistical analysis

Continuous variables are expressed as median (interquartile range) and categorical variables as frequencies and percentages; comparisons used the Mann–Whitney U or Student t-test and the chi-square or Fisher exact test, as appropriate. All tests were two-sided with *p* < 0.05 considered significant. Analyses were performed in Python 3.12 (scikit-learn, XGBoost, LightGBM, CatBoost, SHAP, SciPy, imbalanced-learn) and Stata 14.2; exact versions are listed in Supplementary Table S1.

## 3 Results

### 3.1 Study population

A total of 572 patients hospitalized with AHF were included, of whom 53 (9.3%) died during hospitalization (Figure 1). Baseline characteristics are summarized in Table 1. Compared with survivors, patients who died more often presented with cardiogenic shock, respiratory failure, and acute coronary syndrome, and had higher white-blood-cell counts, blood urea nitrogen, creatinine, NT-proBNP, high-sensitivity troponin I, and aminotransferases, together with lower systolic and diastolic blood pressure (all *p* < 0.05). An acute decompensated heart-failure (ADHF) presentation was markedly less frequent among non-survivors (24.5% vs. 69.7%, *p* < 0.001), reflecting the higher proportion of *de novo* and shock presentations among fatal cases.

**Figure 1:**
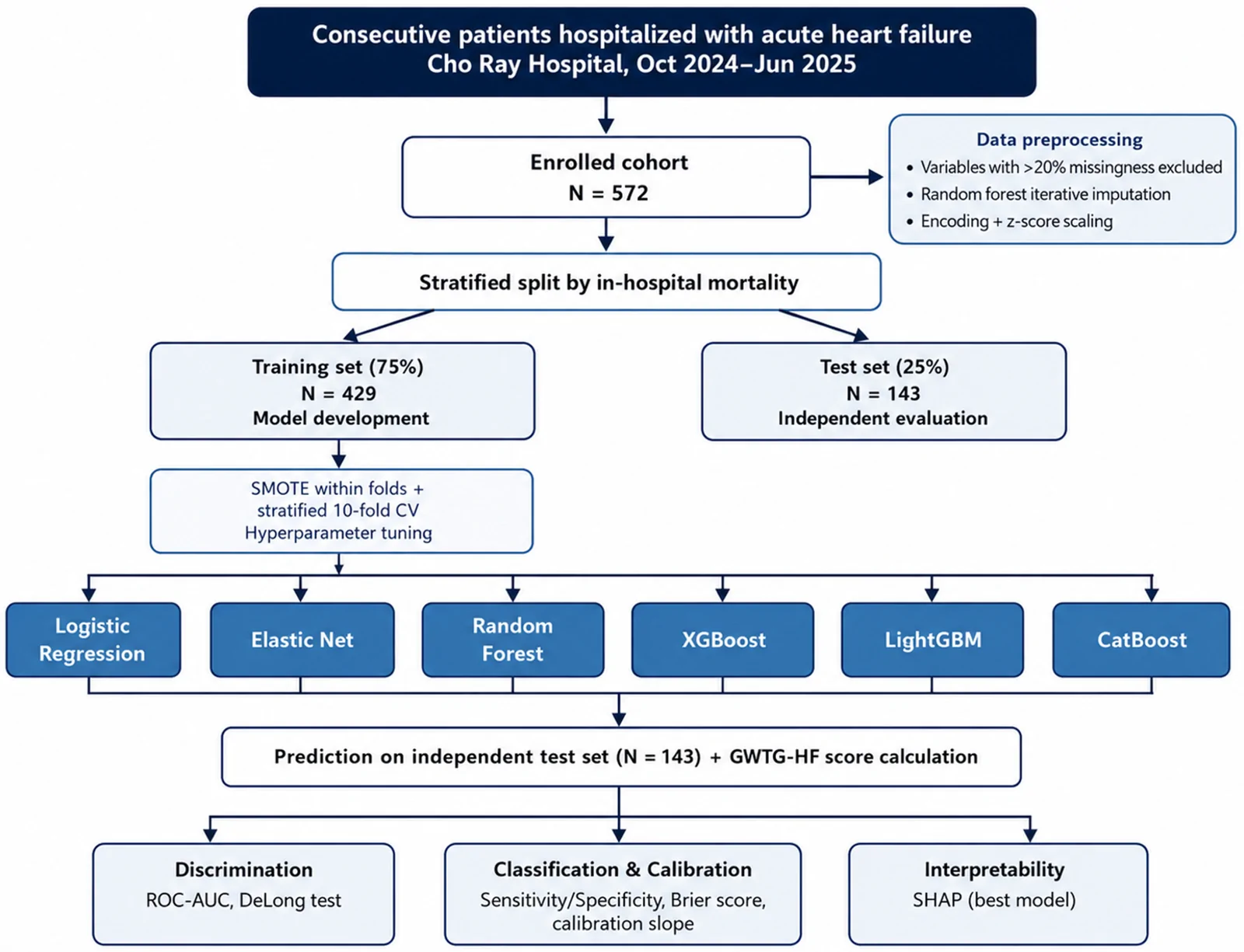
Study flowchart for the development and evaluation of the prediction models. AHF, acute heart failure; CV, cross-validation; GWTG-HF, Get With The Guidelines–Heart Failure; RF, random forest; SHAP, SHapley Additive exPlanations; SMOTE, synthetic minority over-sampling technique.

**Table 1:** Baseline characteristics of the study population.

Variable	All (*n* = 572)	Survivors (*n* = 519)	In-hospital death (*n* = 53)	p	Missing
Demographic characteristics					
Age (years)	64.0 (52.0–72.0)	63.0 (52.0–72.0)	68.0 (55.0–74.0)	0.105	0
Male, *n* (%)	307 (53.7)	277 (53.4)	30 (56.6)	0.760	0
BMI (kg/m2)	22.1 (20.0–24.2)	22.1 (20.1–24.2)	22.4 (19.5–24.4)	0.559	0
Medical history					
Diabetes mellitus, *n* (%)	199 (34.8)	186 (35.8)	13 (24.5)	0.135	0
Hypertension, *n* (%)	337 (58.9)	306 (59.0)	31 (58.5)	1.000	0
Chronic coronary syndrome, *n* (%)	206 (36.0)	190 (36.6)	16 (30.2)	0.437	0
Chronic kidney disease, *n* (%)	114 (19.9)	103 (19.8)	11 (20.8)	1.000	0
Clinical presentation					
Acute decompensated HF, *n* (%)	375 (65.6)	362 (69.7)	13 (24.5)	<0.001	0
Acute pulmonary edema, *n* (%)	109 (19.1)	98 (18.9)	11 (20.8)	0.883	0
Cardiogenic shock, *n* (%)	55 (9.6)	36 (6.9)	19 (35.8)	<0.001	0
Vital signs at admission					
Heart rate (beats/min)	96 (82–110)	96 (82–110)	100 (85–120)	0.279	0
Systolic BP (mmHg)	120 (100–140)	120 (100–140)	100 (80–120)	<0.001	0
Diastolic BP (mmHg)	70 (60–90)	80 (60–90)	60 (50–80)	<0.001	0
Respiratory failure, *n* (%)	306 (53.5)	260 (50.1)	46 (86.8)	<0.001	0
Acute coronary syndrome, *n* (%)	133 (23.3)	109 (21.0)	24 (45.3)	<0.001	0
Electrocardiographic findings					
Atrial fibrillation, *n* (%)	122 (21.3)	113 (21.8)	9 (17.0)	0.525	0
Laboratory findings					
White blood cell count (G/L)	10.1 (7.8–13.9)	9.9 (7.7–13.4)	13.8 (10.2–17.5)	<0.001	0
Hemoglobin (g/L)	118.5 (100–135)	119 (100–135)	109 (95–134)	0.254	0
Platelets (G/L)	232.5 (168–296)	231 (167–295)	251 (172–322)	0.571	0
Sodium (mmol/L)	134 (130–137)	134 (130–137)	133 (130–137)	0.842	0
Potassium (mmol/L)	3.9 (3.4–4.3)	3.8 (3.4–4.2)	4.0 (3.5–4.6)	0.130	0
Blood urea nitrogen (mg/dL)	21.7 (14–33)	21 (14–31)	29 (19–48)	<0.001	0
Creatinine (mg/dL)	1.2 (0.9–1.8)	1.2 (0.9–1.8)	1.5 (1.2–2.3)	<0.001	0
NT-proBNP (pg/mL)	11,716 (4318–29,881)	10,474 (4006–26,691)	26,157 (10,768–35,001)	<0.001	0
NT-proBNP >35,000 pg/mL, *n* (%)	126 (22.0)	107 (20.6)	19 (35.8)	0.018	0
AST (U/L)	45 (30–96)	42 (29–85)	79 (45–249)	<0.001	0
ALT (U/L)	37 (20–80)	35 (20–76)	60 (26–164)	0.004	0
hs-troponin I (ng/mL)	169.7 (44.2–1315.3)	139.9 (41.3–919.5)	2097.9 (282.4–10389.1)	<0.001	25 (4.4)
Glucose (mg/dL)	132 (105–191)	131 (105–185)	168 (113–230)	0.058	16 (2.8)
Echocardiographic findings					
LV dilation, *n* (%)	301 (52.6)	280 (53.9)	21 (39.6)	0.065	0
LVEF (%)	33 (24–46)	33 (24–46)	35 (24–47)	0.914	0
PAPs (mmHg)	45 (34–55)	45 (33–55)	43 (38–58)	0.698	183 (32.0)

Data are median (IQR) or *n* (%). PAPs (>20% missing) was excluded from modeling. ALT, alanine aminotransferase; AST, aspartate aminotransferase; BMI, body mass index; HF, heart failure; LV, left ventricular; LVEF, LV ejection fraction; NT-proBNP, N-terminal pro–B-type natriuretic peptide; PAPs, systolic pulmonary artery pressure.

### 3.2 Candidate predictors and effective sample size

After encoding, 31 candidate predictors entered the models, giving an approximate EPV of 1.7 (1.3 in the development set) (53 events). Multicollinearity was limited after examination of pairwise correlations and variance inflation factors, with the expected correlation between related biomarkers (Supplementary Table S2). Consistent with the low EPV, regularized and shallow tree-based models were prioritized and optimism correction was applied.

### 3.3 Model performance

#### 3.3.1 Discrimination

Discrimination on the independent test set is shown in Figure 2 and Table 2. Random forest achieved the highest ROC-AUC (0.846, 95% CI 0.757–0.922). Logistic regression and elastic net performed comparably (0.795 and 0.794), CatBoost and XGBoost were intermediate (0.786 and 0.763), and LightGBM was lowest (0.694). The GWTG-HF score achieved an AUC of 0.817, exceeding several ML models and numerically comparable to random forest. Because ROC-AUC can be optimistic under class imbalance, we also report the PR-AUC for each model (random forest PR-AUC 0.31; all models in Supplementary Table S4) (29), and optimism-corrected AUCs from repeated cross-validation and bootstrap resampling (random forest optimism-corrected AUC 0.81; Supplementary Table S3), which were modestly lower than the apparent test-set estimates.

**Figure 2:**
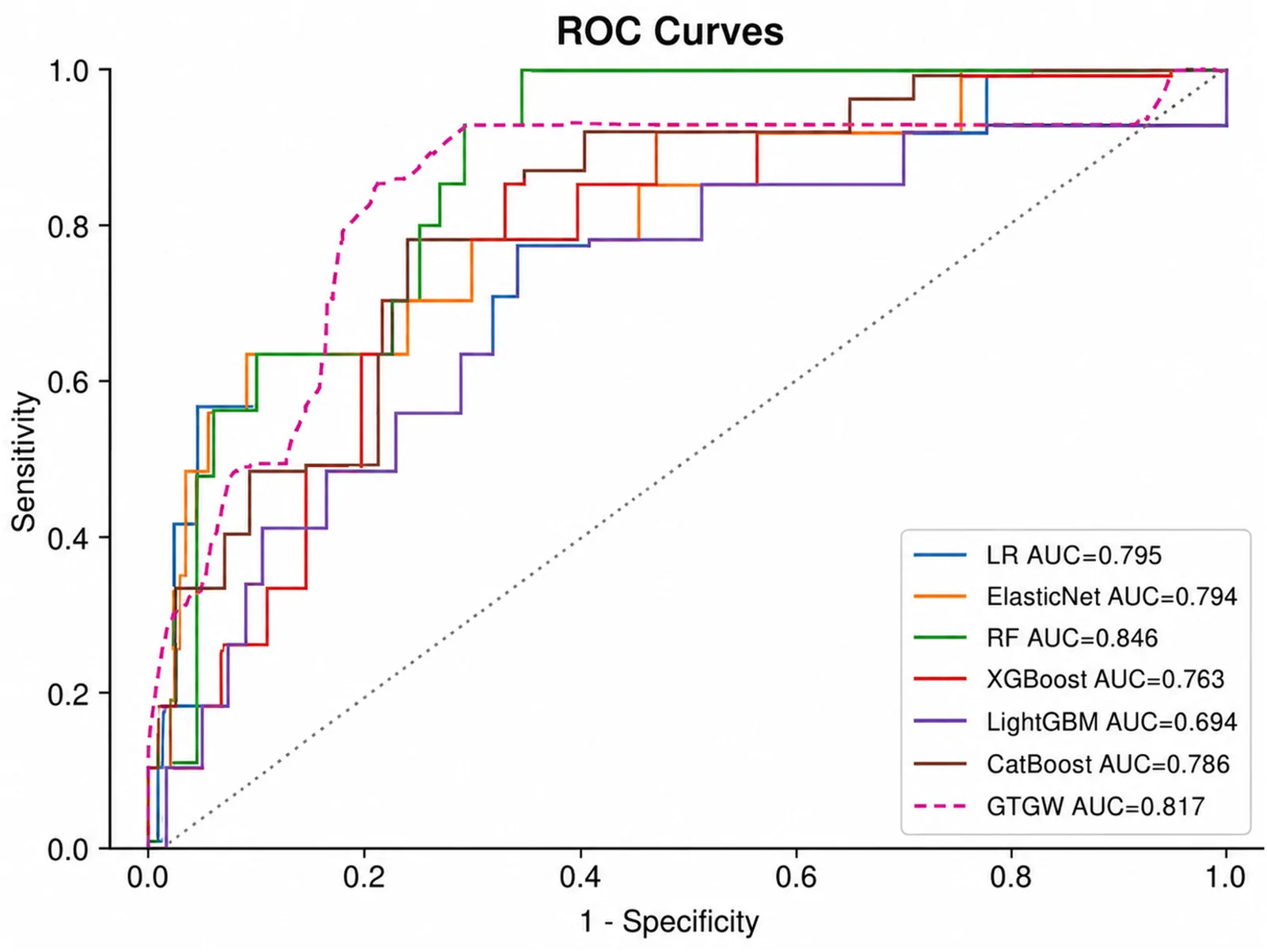
Receiver operating characteristic (ROC) curves for all models on the independent test set. AUC, area under the curve; LR, logistic regression; RF, random forest; GTGW, GWTG-HF score; GWTG-HF, Get With The Guidelines–Heart Failure.

**Table 2:** Discrimination and classification of the machine-learning models and the GWTG-HF score on the independent test set.

Model	Threshold	AUC (95% CI)	PR-AUC	Sensitivity	Specificity	Precision	NPV	F1	F2	Accuracy
Logistic regression	0.12	0.795 (0.653–0.920)	0.32	0.846	0.531	0.153	0.972	0.259	0.444	0.559
Elastic net	0.12	0.794 (0.653–0.918)	0.31	0.846	0.515	0.149	0.971	0.253	0.437	0.545
**Random forest**	**0**.**29**	**0.846** (**0.757–0.922)**	**0**.**31**	**0**.**846**	**0**.**708**	**0**.**224**	**0**.**979**	**0**.**355**	**0**.**545**	**0**.**720**
CatBoost	0.05	0.786 (0.668–0.893)	0.24	0.846	0.592	0.172	0.975	0.286	0.474	0.615
XGBoost	0.09	0.763 (0.636–0.877)	0.24	0.769	0.738	0.227	0.970	0.351	0.521	0.741
LightGBM	0.13	0.694 (0.526–0.847)	0.26	0.538	0.762	0.184	0.943	0.275	0.389	0.741
GWTG-HF score	45.0	0.817 (0.671–0.926)	0.33	0.923	0.677	0.222	0.989	0.358	0.566	0.699

Point estimates are shown; 95% Wilson confidence intervals for sensitivity, specificity, and precision are reported in Supplementary Table S4. PR-AUC values were computed from the reproducible re-analysis pipeline (see Supplementary Methods). Thresholds were derived from training data only. AUC, area under the ROC curve; NPV, negative predictive value; PR-AUC, area under the precision–recall curve.

Bold indicates random forest, the model with the highest ROC-AUC, which was taken forward for SHAP interpretation; bold does not denote the highest value for every metric.

Pairwise DeLong comparisons showed no statistically significant difference between the GWTG-HF score and any ML model (all *p* > 0.05; Table 3). Given only 13 events in the test set, these comparisons were substantially underpowered, and the absence of significance should be interpreted as inconclusive rather than as evidence of equivalent performance.

**Table 3:** DeLong comparison of AUCs between the machine-learning models and the GWTG-HF score.

Model	AUC (ML)	AUC (GWTG-HF)	AUC difference	DeLong p
Logistic regression	0.795	0.817	−0.023	0.840
Elastic net	0.794	0.817	−0.023	0.836
Random forest	0.846	0.817	+0.029	0.713
XGBoost	0.763	0.817	−0.054	0.432
LightGBM	0.694	0.817	−0.123	0.103
CatBoost	0.786	0.817	−0.032	0.629

With only 13 test-set events, these comparisons are underpowered; non-significance does not establish equivalence. ML, machine learning.

#### 3.3.2 Classification performance

Using thresholds derived exclusively from training data, random forest showed the most balanced classification (sensitivity 84.6%, specificity 70.8%, accuracy 72.0%, F2 0.545), and XGBoost the highest accuracy (74.1%) and specificity (73.8%) with acceptable sensitivity (76.9%). For a fair comparison, the GWTG-HF score was evaluated at its threshold with the full set of classification metrics (sensitivity 92.3%, specificity 67.7%, precision 0.222, NPV 0.989, F1 0.358, F2 0.566, accuracy 0.699). Because the test set contained only 13 deaths, all point estimates carry wide confidence intervals (reported in full in Table 2/Supplementary Table S4), and precision was low for every model, reflecting the low outcome prevalence.

#### 3.3.3 Calibration

Calibration is summarized in Table 4 and Supplementary Figure S1. Although CatBoost had the numerically lowest Brier score (0.085) and random forest the calibration slope closest to unity (1.087), the scaled Brier scores (referenced to the no-information Brier score of 0.0826 at the 9.1% test-set prevalence) were low or negative for all models, and the calibration intercepts were consistently negative (from −1.00 to −1.91). Together these indicate systematic over-estimation of risk and limited probabilistic accuracy—an expected consequence of the aggressive imbalance correction (23). The imbalance sensitivity analysis (Supplementary Table S3) confirmed that omitting or reducing over-correction, and applying *post hoc* recalibration, improved the calibration intercept and scaled Brier score while leaving discrimination essentially unchanged. We therefore interpret the models as providing useful risk ranking (discrimination) but requiring recalibration before their predicted probabilities are used for individual decision-making. Calibration of the GWTG-HF score is reported for comparison in Table 4.

**Table 4:** Calibration of the machine-learning models, including the scaled brier score.

Model	Brier score	Scaled Brier	Calibration slope	Calibration intercept
CatBoost	0.085	−0.028	0.556	−1.002
Random forest	0.101	−0.222	1.087	−1.278
XGBoost	0.106	−0.283	0.336	−1.382
LightGBM	0.110	−0.331	0.213	−1.563
Logistic regression	0.143	−0.730	0.500	−1.903
Elastic net	0.144	−0.742	0.503	−1.906
GWTG-HF score	0.073	+0.117	1.633	−0.095

Scaled Brier score (Brier skill score) = 1−Brier/Brier_null_, with Brier_null_ = p(1−p) = 0.0826 at the test-set prevalence (9.1%). Values ≤0 indicate probabilities no better than the no-information rate. A slope closer to 1 and intercept closer to 0 indicate better calibration.

#### 3.3.4 Clinical utility (decision-curve analysis)

Decision-curve analysis across clinically plausible threshold probabilities is shown in Supplementary Figure S2. Across the range most relevant to identifying high-risk AHF patients (approximately 0.05–0.30), both random forest and the GWTG-HF score provided greater net benefit than the treat-all and treat-none strategies, with the GWTG-HF score yielding the highest net benefit across most of this range (31).

### 3.4 Model interpretability (SHAP)

SHAP analysis of the random forest model ranked ADHF presentation, respiratory failure, NT-proBNP, high-sensitivity troponin I, and left ventricular dilation as the most influential features (Figure 3), followed by aspartate aminotransferase, diastolic blood pressure, blood urea nitrogen, cardiogenic shock, and white-blood-cell count. Importantly, the direction of association differed across features: higher NT-proBNP, higher troponin I, respiratory failure, and cardiogenic shock increased the predicted probability of death, whereas the presence of an ADHF phenotype and left ventricular dilation were associated with a lower predicted probability of death. This direction is concordant with the descriptive data (ADHF less frequent among non-survivors) and reflects the higher lethality of *de novo* and shock presentations relative to decompensation of chronic heart failure; it corrects any impression that ADHF is a risk-increasing feature. Overall, the model relied on variables reflecting acute hemodynamic decompensation, myocardial injury, congestion, and end-organ dysfunction.

**Figure 3:**
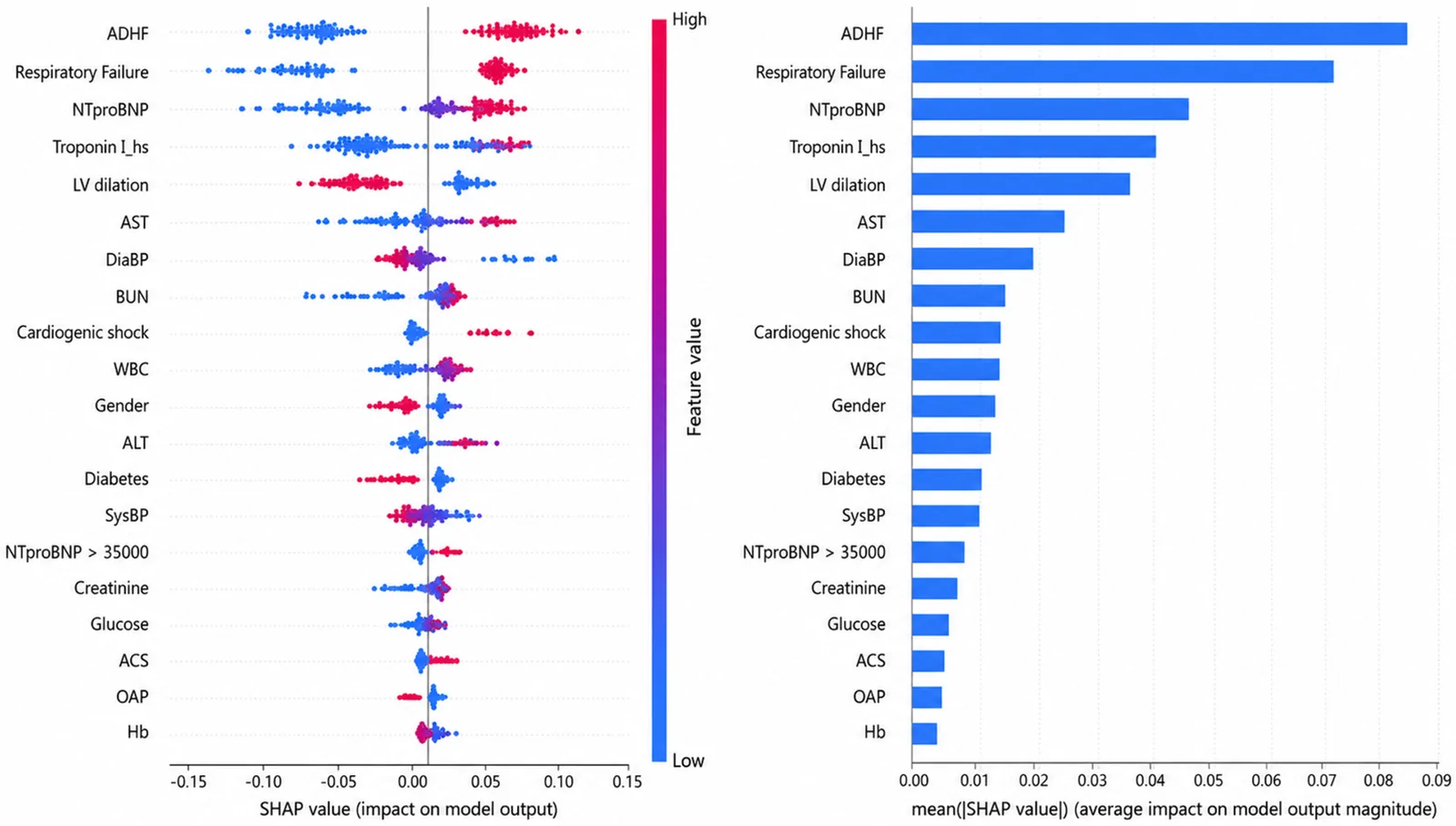
Global SHAP summary for the random forest model. Left, per-patient SHAP values colored by feature value; right, mean absolute SHAP values. Positive SHAP values indicate a higher predicted probability of in-hospital death. ACS, acute coronary syndrome; ADHF, acute decompensated heart failure; ALT, alanine aminotransferase; AST, aspartate aminotransferase; BUN, blood urea nitrogen; DiaBP, diastolic blood pressure; Hb, hemoglobin; LV, left ventricular; NT-proBNP, N-terminal pro–B-type natriuretic peptide; NTproBNP > 35000, NT-proBNP above the assay upper limit (>35,000 pg/mL); OAP, acute pulmonary edema; SysBP, systolic blood pressure; Troponin I_hs, high-sensitivity troponin I; WBC, white blood cell count.

## 4 Discussion

In this prospective cohort of 572 patients hospitalized with AHF, we developed and evaluated six ML models for predicting in-hospital mortality and benchmarked them against the GWTG-HF score. Three findings emerged. First, the ML models achieved good discrimination, with random forest reaching the highest AUC (0.846). Second, no ML model significantly outperformed the GWTG-HF score (AUC 0.817); given only 13 test-set events, this reflects limited statistical power rather than demonstrated equivalence. Third, predicted probabilities were miscalibrated across models, attributable to aggressive imbalance correction—so the models are best regarded as risk-ranking tools pending recalibration, while SHAP provided clinically coherent, direction-resolved interpretation.

### 4.1 Performance of the machine-learning algorithms

Random forest achieved the numerically highest discrimination on the test set, ahead of logistic regression, elastic net, CatBoost, XGBoost, and LightGBM, but these differences were not statistically significant. Probabilistic performance was limited for all models: although CatBoost had the lowest Brier score, the scaled Brier scores were low or negative and the calibration intercepts were uniformly negative, indicating over-estimation of risk. This pattern is the expected consequence of combining SMOTE, class weighting, and scale_pos_weight, and it reinforces the need to report and, where necessary, restore calibration rather than rely on discrimination alone (23, 28). Our findings align with meta-analytic evidence that tree-based ensembles tend to perform well for heart-failure outcomes (14); although XGBoost is often the best model in large ICU-based AHF cohorts (AUCs of 0.821 and 0.816 reported by Li et al. and Tanaka et al.) (33, 34), random forest was more stable in our smaller, prospective cohort.

### 4.2 Machine learning versus the conventional risk score

The GWTG-HF score performed robustly (AUC 0.817), exceeding several ML models and closely approaching random forest, with no significant DeLong difference. This contrasts with reports of clear ML superiority, for example, a deep-learning model (AUC 0.880) outperforming the GWTG-HF score (0.728) (35), and an ML score (AUC 0.90) surpassing established scores (36), but is fully concordant with the systematic review of Christodoulou et al., which found no consistent benefit of ML over logistic regression (16). Crucially, the absence of a significant difference in our cohort most plausibly reflects insufficient power (13 test-set events) rather than genuine equivalence, and should not be read as evidence that ML and GWTG-HF perform identically. The robustness of GWTG-HF nonetheless reaffirms its clinical value in Asian populations (18, 19).

### 4.3 Predictors associated with mortality

SHAP identified ADHF phenotype, respiratory failure, NT-proBNP, high-sensitivity troponin I, and left ventricular dilation as the features most strongly associated with the model output. These are associations learned by the model rather than causal determinants. Whereas the GWTG-HF score relies chiefly on vital signs and renal function (7), the ML model additionally integrated acute presentations and advanced biomarkers, capturing hemodynamic collapse, myocardial injury, and end-organ dysfunction. Elevated NT-proBNP and high-sensitivity troponin are well-validated prognostic markers in AHF (37), and the direction-resolved SHAP analysis clarified that an ADHF phenotype was associated with lower predicted risk, consistent with the greater lethality of *de novo* and shock presentations.

### 4.4 Interpretability, clinical implementation, and deployment

Applying SHAP improved the interpretability of the best-performing model at both global and individual levels, mitigating, though not eliminating, the black-box concern that limits clinical adoption. Any move toward implementation, however, must be tempered. Before an interpretable model such as this could be embedded in electronic health records, several requirements must be met: prospective external and temporal validation; recalibration and ongoing calibration monitoring; specification of the computational and inference requirements (the tree-ensemble models here are lightweight and provide near-instant inference on standard hardware); integration and data-availability constraints at the point of care; clinician usability and alert-fatigue considerations; and applicable regulatory and governance requirements for clinical decision-support software; web-based implementations of ML mortality models have already been demonstrated for intensive-care heart-failure cohorts (38). We therefore frame potential electronic-health-record integration as a future goal contingent on external validation and impact evaluation rather than a present capability.

### 4.5 Strengths and limitations

Strengths include the prospective design, complete outcome ascertainment, rigorous prevention of leakage during imputation and over-sampling, a pre-specified prediction time point, comprehensive evaluation across discrimination, calibration, and clinical utility, and reporting in line with TRIPOD + AI (17). Limitations remain important. First, the number of events was small (53 overall; ∼40 for development, 13 in the test set), yielding a low EPV [1.7 (1.3 in the development set)]; this constrains model stability, inflates the risk of overfitting, and is the most plausible reason the flexible ML models did not significantly exceed GWTG-HF (16, 21). Second, internal validation, even with repeated/nested cross-validation and bootstrap optimism correction, cannot substitute for external validation, which was not feasible in this single-center, nine-month cohort; temporal and multicenter validation are priorities. Third, predicted probabilities were miscalibrated owing to imbalance correction and require recalibration before clinical use. Fourth, classifying all patients as non-Black when computing GWTG-HF, appropriate for this Asian cohort, limits transportability to multi-ethnic populations. Finally, the contribution is a prospective regional clinical validation rather than a methodological advance; demonstrating incremental value beyond GWTG-HF will require larger, multicenter, externally validated studies.

## 5 Conclusion

In a prospective Vietnamese AHF cohort, explainable ML led by random forest achieved good discrimination that was comparable to, but not significantly better than, the established GWTG-HF score, most plausibly reflecting limited statistical power rather than true equivalence. Predicted probabilities were miscalibrated and require recalibration, so the models are best regarded as complementary risk-ranking tools. Direction-resolved SHAP analysis provided transparent, clinically coherent interpretation, highlighting acute presentations, advanced biomarkers, and echocardiographic parameters. Larger multicenter studies with external and temporal validation, recalibration, and prospective impact evaluation are needed before explainable ML can be recommended for routine individualized risk stratification in AHF.

## Data Availability

The raw data supporting the conclusions of this article will be made available by the authors, without undue reservation.
