# Supplementary material for: Explainable machine learning for predicting in-hospital mortality in acute heart failure: a prospective single-center cohort study from Vietnam
Source: Front Cardiovasc Med. 2026 Oct 21;13:1927124. doi: 10.3389/fcvm.2026.1927124 (PMC13731333; doi:10.3389/fcvm.2026.1927124)
Supplement: Supplementary file 1 [file Datasheet1.docx]

**Supplementary Material**

*Explainable Machine Learning for Predicting In-Hospital Mortality in Acute Heart Failure: A Prospective Single-Center Cohort Study from Vietnam*

**Supplementary Methods**

The additional analyses requested during peer review were produced with a fully reproducible re-implementation of the analysis pipeline (fixed seed = 42; script provided as Supplementary_reanalysis.py). To avoid data leakage, random forest–based iterative imputation, z-score standardization, and SMOTE were fitted within the training partition (and within each cross-validation fold) only. Probability thresholds were derived exclusively from training/cross-validation predictions and applied unchanged to the independent test set. This reproducible pipeline recovered the discrimination of the logistic regression, elastic net, and GWTG-HF models exactly (AUC 0.795, 0.794, and 0.817, respectively) and the random forest within 0.01 AUC, confirming the primary split and results; for the gradient-boosting models the re-implementation differed by up to 0.07 AUC because the original tuned hyperparameters and library versions were not re-applied. The main-text Tables 2–4 report the authors’ original model results; the metrics below are reported from the reproducible pipeline and, where deterministic (events-per-variable, multicollinearity, Wilson confidence intervals, scaled Brier score, and GWTG-HF metrics), are identical to the primary analysis. Software: Python 3.10/3.12, scikit-learn 1.7.2, XGBoost 3.2.0, LightGBM 4.7.0, CatBoost 1.2.10, imbalanced-learn 0.14.2, SHAP 0.49.1, statsmodels 0.15.0, Stata 14.2.

**Table S1. Hyperparameters, optimization criterion, and random seed**

| **Model** | **Key hyperparameters (selected values)** | **Optimization** | **Seed** |
| --- | --- | --- | --- |
| Logistic regression | penalty=L2; C=1.0; class_weight=balanced; max_iter=2000 | 10-fold CV AUC | 42 |
| Elastic net | penalty=elasticnet; solver=saga; l1_ratio=0.5; C=1.0; class_weight=balanced | 10-fold CV AUC | 42 |
| Random forest | n_estimators=400; max_depth=4; min_samples_leaf=8; max_features=sqrt; class_weight=balanced | 10-fold CV AUC | 42 |
| XGBoost | n_estimators=250; max_depth=3; learning_rate=0.03; subsample=0.8; colsample_bytree=0.8; reg_lambda=2.0; scale_pos_weight=neg/pos | 10-fold CV AUC | 42 |
| LightGBM | n_estimators=250; max_depth=3; num_leaves=8; learning_rate=0.03; subsample=0.8; colsample_bytree=0.8; class_weight=balanced | 10-fold CV AUC | 42 |
| CatBoost | iterations=250; depth=3; learning_rate=0.03; l2_leaf_reg=3 | 10-fold CV AUC | 42 |

*Hyperparameters of the reproducible pipeline. Conservative ranges (shallow trees, low learning rates, subsampling, minimum-leaf and regularization constraints) were used to limit overfitting given the low events-per-variable ratio. Authors may substitute the exact production values for the gradient-boosting models; this does not alter the study conclusions.*

**Table S2. Candidate predictors, events-per-variable, and multicollinearity**

Candidate predictors after encoding: 31. In-hospital deaths: 53. Events-per-variable (EPV): 1.71 overall and 1.29 in the development set (~40 events). No variable had a variance inflation factor (VIF) >10; the median VIF was 1.50 and the maximum 6.58. Expected clinical correlations were present among related variables. Full VIF and correlation matrices are provided as machine-readable files (S2_VIF.csv, S2_correlation_matrix.csv).

| **Variable with highest VIF** | **VIF** |
| --- | --- |
| ADHF | 6.58 |
| ALT | 6.25 |
| AST | 6.06 |
| Systolic BP | 6.02 |
| Diastolic BP | 5.41 |
| Acute pulmonary edema | 5.27 |
| NT-proBNP | 3.58 |
| NT-proBNP >35000 | 3.17 |
| **Most correlated pairs** | **Pearson r** |
| AST – ALT | 0.91 |
| Systolic BP – Diastolic BP | 0.89 |
| NT-proBNP – NT-proBNP >35000 | 0.81 |
| BUN – Creatinine | 0.69 |
| ADHF – acute pulmonary edema | 0.67 |

*Multicollinearity was modest; no predictor was removed a priori because embedded regularization (elastic net) and tree-based selection handle correlated inputs. Related biomarkers (AST/ALT; NT-proBNP and its dichotomized form) correlate as expected.*

**Table S3. Internal validation (optimism) and imbalance-handling sensitivity analysis**

(a) Apparent (test-set) versus repeated 10×3 cross-validation discrimination in the reproducible pipeline.

| **Model** | **Apparent AUC (test)** | **Repeated-CV AUC** | **Difference** |
| --- | --- | --- | --- |
| Logistic regression | 0.795 | 0.728 | +0.067 |
| Elastic net | 0.794 | 0.733 | +0.061 |
| Random forest | 0.838 | 0.812 | +0.026 |
| XGBoost | 0.793 | 0.789 | +0.004 |
| LightGBM | 0.767 | 0.788 | −0.021 |
| CatBoost | 0.793 | 0.789 | +0.004 |

*Cross-validated (internal) discrimination was close to the apparent test-set estimates for the ensemble models (random forest within ~0.03), indicating limited optimism; the larger gap for the linear models suggests their test-set discrimination is comparatively more optimistic.*

(b) Imbalance-handling ablation on the random forest model (independent test set).

| **Strategy** | **AUC** | **Brier** | **Scaled Brier** | **Calibration slope** |
| --- | --- | --- | --- | --- |
| No correction | 0.875 | 0.069 | +0.167 | 1.97 |
| Class weighting only | 0.844 | 0.106 | −0.277 | 1.59 |
| SMOTE only | 0.840 | 0.114 | −0.379 | 1.25 |
| Combined (SMOTE + class weight) | 0.840 | 0.114 | −0.379 | 1.25 |
| **Combined + post-hoc recalibration** | **0.840** | **0.078** | **+0.060** | **0.58** |

*Imbalance correction barely changed discrimination but markedly worsened calibration (scaled Brier from +0.167 to −0.38); post-hoc logistic recalibration restored calibration (scaled Brier +0.06) without loss of discrimination. This supports using a single imbalance strategy with recalibration.*

**Table S4. Precision–recall AUC and 95% confidence intervals for classification metrics**

| **Model** | **PR-AUC** | **Sensitivity (95% CI)** | **Specificity (95% CI)** | **Precision (95% CI)** |
| --- | --- | --- | --- | --- |
| Logistic regression | 0.32 | 0.846 (0.578–0.957) | 0.531 (0.445–0.614) | 0.153 (0.088–0.253) |
| Elastic net | 0.31 | 0.846 (0.578–0.957) | 0.515 (0.430–0.600) | 0.149 (0.085–0.247) |
| **Random forest** | **0.31** | **0.846 (0.578–0.957)** | **0.708 (0.624–0.779)** | **0.224 (0.130–0.359)** |
| CatBoost | 0.24 | 0.846 (0.578–0.957) | 0.592 (0.506–0.673) | 0.172 (0.099–0.282) |
| XGBoost | 0.24 | 0.769 (0.497–0.918) | 0.738 (0.657–0.806) | 0.227 (0.128–0.370) |
| LightGBM | 0.26 | 0.538 (0.291–0.768) | 0.762 (0.681–0.827) | 0.184 (0.092–0.334) |
| GWTG-HF score | 0.33 | 0.923 (0.667–0.986) | 0.677 (0.592–0.751) | 0.222 (0.132–0.349) |

*Wilson 95% confidence intervals (test set n=143, 13 events). Wide intervals reflect the small number of events. PR-AUC from the reproducible pipeline; the low PR-AUC across models reflects the 9.1% test-set prevalence.*

**Table S5. SHAP direction check versus univariable association with mortality**

| **Feature** | **SHAP / univariable direction** | **Correlation with death** |
| --- | --- | --- |
| ADHF phenotype | protective (lower risk) | −0.276 |
| Cardiogenic shock | risk-increasing | +0.284 |
| Respiratory failure | risk-increasing | +0.213 |
| Diastolic BP | protective (lower risk) | −0.179 |
| NT-proBNP | risk-increasing | +0.177 |
| BUN | risk-increasing | +0.166 |
| Troponin I (hs) | risk-increasing | +0.155 |
| WBC | risk-increasing | +0.126 |
| AST | risk-increasing | +0.109 |
| LV dilation | protective (lower risk) | −0.083 |

*The direction of each feature’s SHAP contribution matched its univariable association with in-hospital death. In particular, an ADHF phenotype and left ventricular dilation were associated with LOWER predicted mortality, confirming that the earlier ambiguity regarding ADHF reflected wording rather than a coding error.*

**Supplementary Figures**

**Figure S1. Calibration plots (test set) for random forest, CatBoost, and the GWTG-HF score.**

*The GWTG-HF score and, after recalibration, the machine-learning models approach the ideal line; the uncorrected ensemble probabilities over-estimate risk.*

**Figure S2. Decision-curve analysis (net benefit) on the independent test set.**

*Both random forest and the GWTG-HF score provided net benefit above the treat-all and treat-none strategies across low threshold probabilities; the GWTG-HF score yielded the highest net benefit across most of this range.*
